# Supplementary material for: High circulating fibroblast growth factor-21 levels as a screening marker in fatty pancreas patients
Source: PeerJ. 2023 Apr 12;11:e15176. doi: 10.7717/peerj.15176 (PMC10105565; doi:10.7717/peerj.15176)
Supplement: Supplemental Information 1 [file peerj-11-15176-s001.docx]

**Supplementary Table 1** The levels of serum FGF21 and adipocytokine levels in male and female with FP.

| **Variables** | **Male (N=58)** | **Female (N=41)** | ***P* Value** |
| --- | --- | --- | --- |
| FGF-21  Leptin  Resistin  Adiponectin  TNF-α | 366.74±204.26  3.37(1.60,6.88)  8.07±4.23  7.55±4.63  55.52±3.79 | 266.82±134.47  7.14 (2.65,23.06)  9.08±4.39  8.37±5.26  58.05±4.84 | 0.004^**^  0.001^**^  0.253  0.414  0.004^**^ |

S Table 2: FP, fatty pancreas; FGF-21, fibroblast growth factor-21; TNF-α, tumor Necrosis Factor-alpha. ^**^P<0.01.
